# A novel methylation marker on the *CLEC14A* gene for cervical cancer screening

**DOI:** 10.3389/fonc.2026.1730952

**Published:** 2026-04-14

**Authors:** Sixian Wang, Hao Xiao, Huafang Gao

**Affiliations:** 1 Graduate School of Peking Union Medical College, Beijing, China; 2 National Research Institute for Family Planning, Beijing, China; 3 Beijing Zhipu Medical Laboratory Co., Ltd., Beijing, China

**Keywords:** biomarker, cervical cancer, cg05057720, CLEC14a, DNA methylation

## Abstract

**Introduction:**

Despite advancements in cervical cancer early detection, such as HPV screening, their inherent limitations, such as unsatisfactory specificity, necessitate the discovery of novel, highly accurate biomarkers for cervical cancer and its precancerous high-grade squamous intraepithelial lesions (HSILs).

**Methods:**

In this study, we identified a novel methylation marker, the cg05057720 site within the *CLEC14A* gene, through an initial analysis of The Cancer Genome Atlas (TCGA) and independent validation in the GSE46306 dataset. The marker’s clinical utility was further evaluated using the Quantitative Amplification of Specific Methylation (QASM) assay across a comprehensive cohort of 431 participants, including normal (n=130), LSIL (n=83), HSIL (n=195), and tumor samples (n=23).

**Results:**

We observed a progressive increase in *CLEC14A* cg05057720 methylation correlating with lesion severity, where median levels were 0% in normal samples, 2.9% in LSIL, 25.7% in HSIL, and 48.6% in tumors. In receiver-operating characteristic (ROC) analysis, the marker demonstrated exceptional diagnostic performance, achieving a sensitivity of 100% and a specificity of 95.3% for the detection of HSIL and invasive cancer in the validation cohort.

**Discussion:**

This proof-of-concept work establishes *CLEC14A* cg05057720 as a sensitive and specific epigenetic biomarker, providing a robust theoretical basis for utilizing this marker to improve risk stratification and reduce unnecessary tests and treatments, though future large-scale multi-center prospective validations are warranted to further confirm these findings.

## Introduction

Cervical cancer is one of the most common gynecological tumors, ranking fourth among female cancers. In 2020, there were 604,000 new cases and 342,000 deaths globally (1). The incidence of cervical cancer is rising worldwide, with predictions of 700,000 new cases and 400,000 deaths by 2030 (2).

A multistep carcinogenesis model has been established and is well accepted (3), with HPV infection, progression to precancer, and invasion to cancer viewed as critical steps. Tumorigenesis of cervical cancer typically begins with persistent infection by high-risk human papillomavirus (hrHPV), which leads to precancerous lesions, including grade 2 or 3 cervical intraepithelial neoplasia (CIN2/CIN3). High-grade lesions have the potential to progress into cervical cancer, with approximately 30% of CIN3 cases evolving into cancer within 30 years (4). CIN is also referred to as squamous intraepithelial lesion (SIL), with low-grade lesions (low-grade SIL, LSIL) corresponding to CIN1, and high-grade lesions (high-grade SIL, HSIL) corresponding to CIN2/3 (5). The progression from CIN to invasive carcinoma is a slow process with a high likelihood of regression. Among patients with CIN 1, 60% experience regression or disappearance, whereas 43% of those with CIN 2 and 32% of those with CIN 3 exhibit regression or disappearance (6, 7). HSIL carries a risk of developing into cervical cancer and is referred to as a precancerous lesion. Despite the availability of effective prophylactic HPV vaccines and organized screening programs utilizing cytology and HPV DNA testing, disparities in access and implementation persist, particularly in low-resource settings, leading to late-stage diagnoses and poorer prognoses (8). Early detection and accurate risk stratification are, therefore, critical for improving patient outcomes and reducing mortality rates.

Current cervical screening methods, including cytology-based methods and HPV screening, have limitations. Cytology-based screening (e.g., Pap test) suffers from variable sensitivity and specificity, leading to false negatives and positives, respectively. While HPV DNA testing offers higher sensitivity, it has lower specificity, particularly in younger women, where transient HPV infections (9), are common. The identification of precancerous lesions, specifically high-grade cervical intraepithelial neoplasia (CIN2/3), is crucial as these lesions are direct precursors to invasive cancer. Thus, there is a pressing need for more precise biomarkers to identify cervical cancer and high-risk precancerous lesions.

In addition to high-risk human papillomavirus (hrHPV) infection, recent research has shown that epigenetic modifications, particularly DNA methylation, play a crucial role in regulating gene expression and are frequently dysregulated in cancer (10, 11). As for cervical cancer, the methylation levels of particular genes are associated with the severity of cervical lesions and the advancement of high-grade cervical intraepithelial neoplasia (12, 13), demonstrating its prognostic potential. It has been reported that changes in methylation of host or viral genes serve as valuable early detection biomarkers, used for triaging HPV-positive patients or determining whether patients with abnormal cytology are at risk of high-grade lesions (14–16). This method, however, relied on the methylation level of HPV genes, limiting it to detecting HPV^-^ cervical cancers. Meanwhile, compared with multi-panel, single-gene assays showed a cost and time advantage. Nonetheless, the report of a single, non-HPV methylation marker for cervical cancer screening is still lacking.

In the present work, we discovered that cervical cancer can be characterized by the methylation at the cg05057720 site of the *CLEC14A* locus by analyzing TCGA datasets. Moreover, its utility was validated using clinical samples, confirming that the methylation site of the *CLEC14A* gene can serve as a novel biomarker for screening high-grade cervical lesions or tumors sensitively and precisely. Our work presented it as a promising candidate for improving cervical cancer diagnostics.

## Materials and methods

### Public data analysis

The cancer genome atlas (TCGA) dataset used was TCGA-CESC, obtained from https://portal.gdc.cancer.gov/. The methylation microarray (HM450) detection data and RNA-Seq detection data were analyzed.

The GSE46306 dataset was obtained from Gene Expression Omnibus (GEO, https://www.ncbi.nlm.nih.gov/geo/), and the processed data were downloaded and analyzed.

### Clinical specimen

All procedures performed in studies involving human participants were in accordance with the ethical standards of the institutional and/or national research committee and with the 1964 Helsinki Declaration and its later amendments or comparable ethical standards. The study was approved by the institution’s ethics committee under approval number 2016sz-007. Exfoliated cervical cell specimens were suspended in a preservative solution (Zensun Biotech, Xiamen, China) and stored at 4°C. All samples were obtained from Beijing Zhipu Medical Laboratory Co., Ltd., and all patients signed informed consent forms. The clinical classification of all specimens was confirmed by histopathological examinations, which served as the gold standard. For this study, low-grade squamous intraepithelial lesions (LSIL) correspond to histologically confirmed CIN1, while high-grade squamous intraepithelial lesions (HSIL) correspond to histologically confirmed CIN2 and CIN3.

### DNA extraction and bisulfite treatment

The genomic DNA was extracted from cervical exfoliated cells according to the instructions of the Tiangen genomic DNA Kit (Tiangen Biotech, Beijing, China). The concentration of DNA was measured using a micro-volume UV spectrophotometer (Orion Nano100, Hangzhou Aosheng Instruments, China).

The DNA, after extraction, was processed with bisulfite using the DNA Bisulfite Conversion Kit (DP215, Tiangen Biotech), and it was then applied to the next stage of sequencing.

### Targeted DNA methylation sequencing

The PCR reaction was conducted utilizing the Methylation-specific PCR kit (Tiangen Biotech, Beijing, China). Genomic DNA was subjected to sodium bisulfite conversion as previously described. For bisulfite sequencing, target regions were amplified using bis-nCLE (bisulfite-non-CpG-Large-Elimination) primers (bis-nCLE-F/R). See primers used for amplification in **Supplementary Data S1**. The PCR volume was 20 µL with 1µL bis-nCLE-F primer (10µM), 1µL bis-nCLE-R primer (10µM), 0.2µL 10×MSP PCR Buffer, 0.6µL dNTPs (2.5mM), 0.4µL MSP DNA Polymerase (1.5U/µL), 12µL deionized water, and 2 µL bisulfite-modified DNA. The PCR program comprised an initial denaturation step at 95°C for 5 minutes, followed by 40 cycles (94°C for 20 seconds, 55°C for 30 seconds, and 72°C for 20 seconds), and concluded with a final extension at 72°C for 20 seconds. The PCR products were subsequently sent to the sequencing service provider (Beijing Tianyi Huiyuan Biotechnology Co., Ltd.) for sequencing.

### Methylation-specific fluorescence quantitative PCR detection

We performed quantitative analysis of single-base methylation (QASM) as previously described (17). Briefly, genomic DNA was first subjected to sodium bisulfite treatment. Post conversion, a single-tube competitive PCR was performed using a pair of allele-specific TaqMan-MGB probes (See primers used for amplification in **Supplementary Data S1**). The probes were designed to overlap the same single CpG site: a 6FAM-labeled probe specific to the methylated (C) allele and a VIC-labeled probe specific to the unmethylated (T) allele. To achieve high single-base discrimination, both probes incorporated a Minor Groove Binder (MGB) at the 3’ end. This dual-probe quantification and normalization strategy was adapted from previously described methods (18, 19). The 20 µL reaction mixture contained 400 ng of bisulfite-converted DNA, 500 nmol/L of each primer, 300 nmol/L of each MGB-probe, and 1× Superreal Premix (Tiangen Biotech, Beijing, China). Amplification was performed on the ABI 7500 system (Thermo Fisher Scientific) with an initial denaturation at 95°C for 15 min, followed by 45 cycles of 94°C for 20 s and 58°C for 40 s. The methylation percentage was calculated using the formula 1/(1 + 1/2^-ΔCT^)×100%, ΔCT=CT_methylation_- CT_unmethylation_ (20).

### ROC analysis

Firstly, the entire QASM dataset was split into a training set (Normal n=91, LSIL n=58, HSIL n=136, cancer n=16) and a validation set (normal n=39, LSIL n=25, HSIL n=59, cancer n=7). The split processed was performed using Microsoft Excel. Briefly, for each group, including normal, LISL, HSIL, and cancer, random labels were generated using the *rand* function and were assigned to each sample in the group, as an additional column. The data in each group was then ranked in descending order for data shuffling. After that, the first 70% in each group was assigned to the training set, while the remaining 30% was assigned to the validation set.

After the training/validation-set split, the ROC curve analysis was then conducted utilizing IBM SPSS Statistics 22.0 (IBM Corporation, Armonk, NY, USA). Briefly, in the training set, the optimal methylation cutoff for distinguishing the target group from the control group was determined by maximizing the Youden Index. This pre-defined cutoff was then applied to the validation set to assess the marker’s sensitivity, specificity, and Area Under the Curve (AUC).

### Statistical analyses

We used GraphPad Prism 10.1.0 for all experimental data analysis. For pairwise comparisons, we employed two-tailed unpaired Student’s t-tests. For multi-group comparisons, we utilized the Mann-Whitney test for pairwise comparisons. The specific statistical test for each experiment is noted in its corresponding figure legend. Continuous data are presented as median ± SEM or mean ± SEM. A p-value < 0.05 was deemed statistically significant, with significance levels indicated by asterisks: *p < 0.05, **p < 0.01, ***p < 0.001, ****p < 0.0001.

## Result

### Identification of *CLEC14A* cg05057720 hypermethylation in cervical cancer

**Figure 1:**
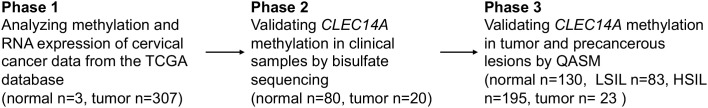
Study design. Schematic diagram showing the workflow.

**Figure 2:**
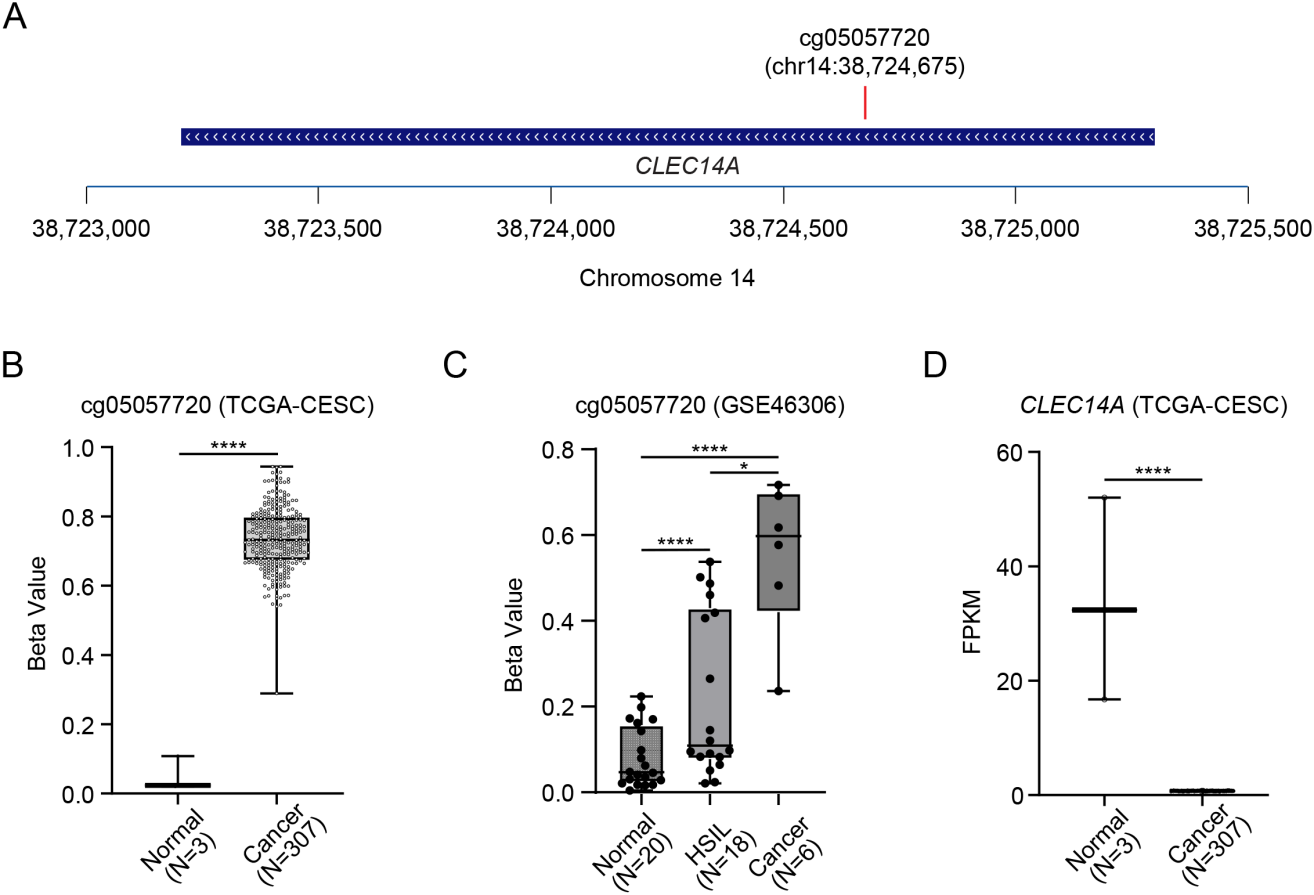
*CLEC14A* cg05057720 hypermethylation is a hallmark of cervical cancer. **(A)** Schematic representation of the CLEC14A gene locus. The position of the CpG site cg05057720 (chr14:38,724,675) within the gene body is indicated by the red vertical line. **(B)** Methylation status of (beta value) *CLEC14A* cg05057720 in normal and tumor tissues (data source: TCGA-CESC). **(C)** Methylation status of (beta value) *CLEC14A* cg05057720 in normal, HSIL (CIN3), and tumor tissues (data source: GSE46306). **(D)** RNA expression of *CELC14A* (fragments per kilobase of exon per million mapped reads, FPKM) in normal and tumor tissues. Data in **(B-D)** are presented as mean ± SEM. Statistical analyses Student’s t-test, *p < 0.05, ****p < 0.0001.

To systematically identify aberrantly methylated genes implicated in cervical tumorigenesis, we initiated our investigation by leveraging a publicly available dataset from The Cancer Genome Atlas (TCGA), which provided comprehensive methylation and RNA expression profiles from 307 cervical cancer samples alongside 3 normal tissue samples (**Figure 1**). The results revealed a specific methylation site, cg05057720, situated within the *CLEC14A* gene (**Figure 2A**), that exhibited significantly elevated methylation levels in tumor samples compared with normal tissue controls. Quantitatively, the cg05057720 hypermethylation was markedly pronounced in cancerous samples, demonstrating a mean Δbeta value of 0.69 in tumor tissues compared with 0.024 in normal tissues (**Figure 2B**). However, TCGA datasets, including the CESC cohort, are frequently characterized by a limited number of normal tissue controls (n=3 in this study) (21), which constrained the statistical power of comparisons. To further corroborate this finding, we performed a secondary validation using an independent dataset (GSE46306), which provided a more balanced distribution of normal tissues (n=20). Consistent with the TCGA results, cg05057720 methylation levels showed a progressive and significant increase from normal tissues to HSIL (n=18) and reached the highest levels in invasive cancer (n=6) (**Figure 2C**).

Given the well-established role of DNA methylation in regulating gene expression (22), we subsequently explored the functional consequence of this observed hypermethylation on *CLEC14A* gene expression. Our findings consistently showed a significant downregulation of the CLEC14A gene within tumor tissues compared to its expression in normal tissues (**Figure 2D**). This inverse correlation between cg05057720 hypermethylation and *CLEC14A* gene expression suggests that epigenetic silencing via methylation may be a hallmark of cervical cancer.

### Hypermethylation of CLEC14A cg05057720 was validated using clinical samples

**Figure 3:**
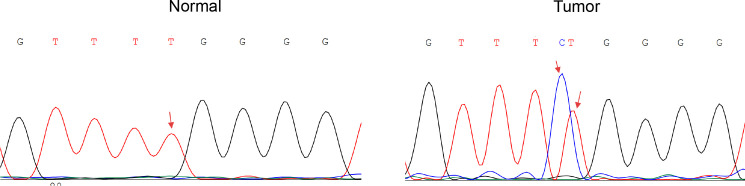
Representative sanger sequencing chromatograms. representative bisulfite sequencing chromatograms illustrating methylation patterns at a specific CpG site in tumor and normal tissues. The arrows highlight the analyzed CpG site.

Following the promising in silico findings, we next validated cg05057720 as a biomarker for cervical cancer using an independent set of clinical samples. For this purpose, we performed targeted Sanger bisulfite sequencing on a cohort of 100 cervical exfoliated cell samples (**Figure 1**). This cohort comprised 20 histologically confirmed cervical cancer samples and 80 normal samples (**Figure 1**, **Table 1**). Strikingly, while complete methylation at the cg05057720 site within the *CLEC14A* locus was unequivocally detected in all 20 (100%) cancer samples, no methylation was observed at this specific site in any of the 80 normal samples (**Figure 3**). This distinction between cancer and normal samples in our validation cohort highlights the accuracy and precision of *CLEC14A* cg05057720 to differentiate malignant from healthy cervical tissues.

**Table 1:** Demographic table.

Characteristic	Bisulfite sequencing cohort (n=100)	QASM cohort (n=431)
Age (years)		
Mean ± SD	46.6± 9.1	44 ± 12.4
Range	32-66	20-67
Histological Type		
Squamous Cell Carcinoma	18 (18%)	20 (4.6%)
Adenocarcinoma	2 (2%)	3 (0.7%)
HISL (CIN2/3)	/	195(45.2%)
LSIL (CIN1)	/	83(19.3%)
Normal	80(80%)	130(30.2%)

### Methylation level of *CLEC14A* cg05057720 increases during cervical cancer tumorigenesis

Recognizing the clinical importance of early detection and the progressive nature of cervical carcinogenesis, as SILs can advance to invasive tumors. We next extended our investigation to assess the methylation level of *CLEC14A* cg05057720 across different stages of cervical lesion progression. We conducted quantitative analysis of single-base methylation (QASM) (17) on an additional, larger cohort of 431 cervical exfoliated samples (**Figure 1**), categorized into different stages based on histology, including 23 cervical cancer cases, 130 normal samples, 195 high-grade squamous intraepithelial lesion (HSIL) samples, and 83 low-grade squamous intraepithelial lesion (LSIL) samples (**Table 1**).

**Figure 4:**
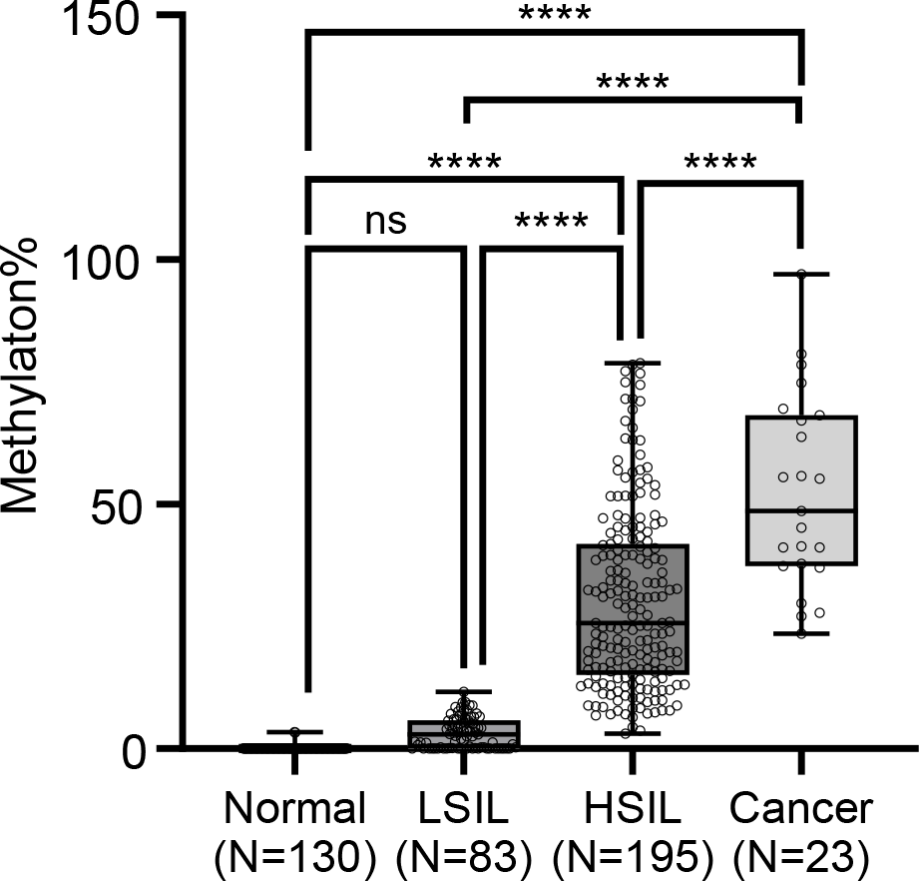
Progressive increase in *CLEC14A* cg05057720 methylation levels during cervical cancer tumorigenesis. Boxplot representing the rate of methylation in the *CLEC14A* cg05057720 site across distinct groups or conditions: normal tissue (n=130), LSIL lesions (histopathology CIN1, n=83), HSIL lesions (histopathology CIN2/3, n=195), and cervical cancer tissues (n=23). Data are presented as mean ± SEM. Statistical analyses by Mann-Whitney analysis test, ****p < 0.0001.

Our analysis revealed a consistent pattern in the methylation levels at *CLEC14A* cg05057720 and cervical cancer tumorogenesis. Specifically, the median methylation levels were 0% in normal samples (range: 0–3.4%) and 2.9% in LSIL (range: 0–11.6%), showing no statistical significance in this specific trend (**Figure 4**, **Table 2**). In contrast, we observed a significant elevation of methylation in HSIL (median: 25.7%, range: 3.1–78.8%), which reached its highest levels in invasive tumor samples (median: 48.6%, range: 23.5–97.0%) (**Figure 4**, **Table 2**). These results suggest that *CLEC14A* cg05057720 methylation is not merely a marker of established cancer but rather an early and accumulating epigenetic event in cervical tumorigenesis, making it a promising candidate for identifying high-risk precancerous stages.

**Table 2:** QASM analysis of CLEC14A cg05057720 across groups.

Characteristic	Normal	LSIL	HSIL (CIN2/3)	Cancer
Number	130	83	195	23
Range	0-3.4%	0-11.6%	3.1%-78.8%	23.5-97%
median	0%	2.9%	25.7%	48.6%
P-value	0.5	<0.0001	<0.0001	<0.0001

### *CLEC14A* cg05057720 is a reliable marker to identify cervical cancer and high-risk precancerous lesions

**Figure 5:**
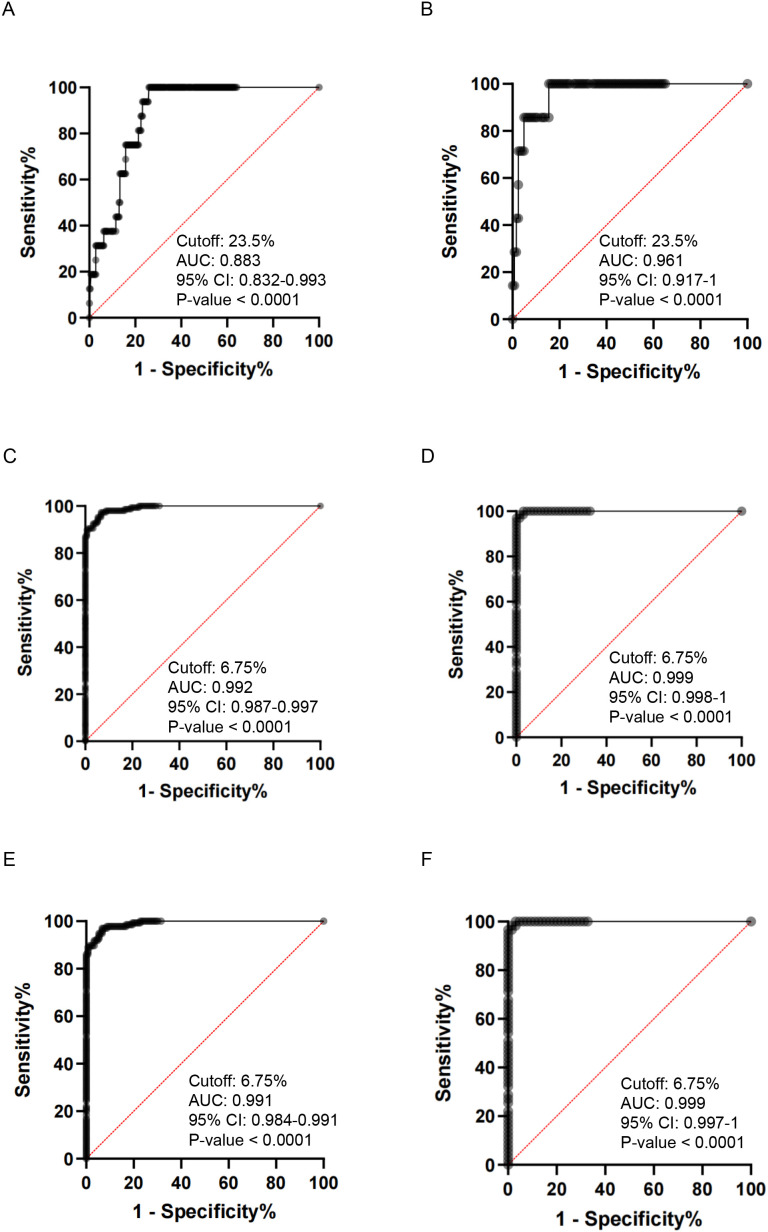
Diagnostic Performance of *CLEC14A* cg05057720 Methylation as a Biomarker. ROC curves illustrate the diagnostic performance of the methylation biomarker in discriminating different clinical stages. **(A, B)** Discrimination of invasive cervical cancer from the combined group of HSIL (histopathology CIN2/3), LSIL, and normal tissues. **(C, D)** Discrimination of HSIL+ (Cancer and HSIL) from LSIL and normal tissues. **(E, F)** Discrimination of HSIL from LSIL and normal tissues. Panels on the left **(A, C, E)** represent the training set (Normal n=91, LSIL n=58, HSIL n=136, Cancer n=16), while panels on the right **(B, D, F)** represent the validation set (Normal n=39, LSIL n=25, HSIL n=59, Cancer n=7). Annotated data within each plot indicates the optimal methylation cutoff, Area Under the Curve (AUC), 95% Confidence Interval (CI), and P-value. See the matrices also in **Table 2**.

To quantitatively assess the diagnostic performance of *CLEC14A* cg05057720, we first analyzed its ability to discriminate invasive cervical cancer from the non-cancerous ones, including HSIL, LSIL, and normal tissues. Using a methylation threshold of 23.5% as the cutoff value, the area under the curve (AUC) for distinguishing malignant samples was 0.883 (95% CI: 0.832–0.933) in the training set (**Figure 5A**) and increased to 0.961 (95% CI: 0.917–1.000) in the validation set (**Figure 5B**). Notably, the marker achieved a sensitivity of 100% and a specificity of 84.6% in the validation cohort (**Table 3**), though we acknowledge that the total number of cancer samples (n=16 training; n=7 validation) is small.

**Table 3:** ROC analysis and diagnostic accuracy metrics.

Detection target	Group	AUC	Sensitivity	Specificity	Cut-off
Cancer (vs. HSIL/LSIL/Normal)	Training (n=301)	0.883	100%	74.0%	23.5%
	Validation (n=130)	0.961	100%	72.4%	23.5%
HSIL+ (Cancer+HSIL vs. LSIL/Normal)	Training (n=301)	0.992	97.3%	93.3%	6.75%
	Validation (n=130)	0.999	100%	95.3%	6.75%
HSIL (vs. LSIL/Normal)	Training (n=285)	0.991	97.1%	93.3%	6.75%
	Validation (n=123)	0.999	100%	95.3%	6.75%

Given that malignancies are commonly rare in screening populations, we secondly expanded our analysis to assess the marker’s utility in identifying the broader category of clinically significant lesions (HSIL+, encompassing both HSIL and tumors), which evaluates the marker’s overall power in a triage setting. Utilizing an optimized threshold of 6.75%, the AUC remained high at 0.992 (95% CI: 0.987–0.997) in the training set (**Figure 5C**) and reached a near-perfect 0.999 (95% CI: 0.998–1.000) in the validation set (**Figure 5D**). Quantitatively, the marker demonstrated a sensitivity of 100% and a specificity of 95.3% in the validation set for detecting HSIL+ (**Table 3**). Interestingly, the optimal threshold for distinguishing HSIL versus LSIL/normal was also found to be 6.75%, resulting in a similar AUC of 0.991 (95% CI: 0.984–0.998) in the training cohort (**Figure 5E**) and 0.999 (95% CI: 0.997–1.000) in the validation cohort (**Figure 5F**). Similarly, to identifying HSIL+ cases, the 6.75% cutoff could distinguish HSIL lesions from LSIL and normal samples with 100% sensitivity and 95.3% specificity. Worth mentioning, this identical cutoff (6.75%) and similar metrics for both HSIL and HSIL+ likely reflect the robust methylation signal present in high-grade lesions, though it may also be influenced by the relatively small cancer sample size (**Table 1**).

Taken together, these results establish *CLEC14A* cg05057720 as a highly sensitive and specific epigenetic marker. Its progressive performance demonstrates its potential as an objective method to identify patients who truly require colposcopy, thereby reducing the burden of unnecessary follow-up for low-grade cases, even though larger multi-center prospective studies are required to further validate its diagnostic efficacy and generalizability across diverse screening populations.

## Discussion

In the present study, we identify *CLEC14A* cg05057720 hypermethylation as a novel and highly promising epigenetic biomarker for the detection and risk stratification of cervical cancer and HSILs. We demonstrated that methylation levels at this specific CpG site progressively increase with the severity of cervical lesions, from normal samples through LSIL and HSIL, culminating in the highest levels in invasive cervical cancer. This progressive epigenetic signature is reflected in the marker’s robust diagnostic performance across all high-grade categories in our validation cohort.

CLEC14A is a transmembrane protein that is expressed primarily on endothelial cells and participates in cell-cell adhesion and angiogenesis (23, 24). CLEC14A appears to exert context-dependent effects on tumorigenesis, likely due to its dual roles in suppressing cell-cell adhesion and promoting angiogenesis (25, 26). While findings from lung adenocarcinoma (LUAD) link *CLEC14A* hypermethylation and downregulation to cancer progression (27), overexpression has been reported in hepatocellular carcinoma (HCC) (28). Such discrepancies necessitate further investigation into the tissue-specific roles of *CLEC14A* methylation and expression.

This work also yields several limitations. Foremost, the interpretation of our findings is constrained by the single-center, retrospective case-control design. Single-center studies lack external validity that ensures our marker’s generalizability to broader populations and clinical resources such as nurse/patient ratios (29). Additionally, our study is limited by the insufficient validation of the marker in adenocarcinoma, as such samples were underrepresented in our cohort (**Table 1**). Given that squamous cell carcinoma and adenocarcinoma of the cervix possess different molecular and immunological patterns (30), whether our methylation marker could perform equally well in the two subtypes warrants further investigations that include sufficient adenocarcinoma samples. Moreover, the risk of overfitting in our ROC analysis cannot be neglected as well due to our single-centered cohort with a relatively limited cohort size. Last but not least, the lack of prospective cohort studies hindered follow-up investigations (31), especially into whether the methylation level of *CLEC14A* progressively increases during the LSIL-to-tumor transition.

In addition to improved cohort design, future studies are also required to investigate the exact mechanism of how *CLEC14A* hypermethylation and subsequently decreased expression contribute to cervical cancer tumorogenesis demands further exploration, especially when opposite expression patterns were observed in HCC models. Moreover, although this biomarker showed sufficient precision and accuracy in cervical samples, further investigations are needed to explore its compatibility with less invasive sample types. For instance, exploring whether this cg05057720 hypermethylation pattern can be reliably detected in circulating tumor DNA (ctDNA) would significantly enhance its clinical utility, offering a more convenient and patient-friendly screening method.

It is also essential to benchmark *CLEC14A* methylation against the current leading alternatives that were detailedly reviewed by Sumiec et al. (32). Firstly, it is important to note, however, that while these metrics are promising, our performance data are derived from a retrospective, single-center study. The p16/Ki-67 dual stain, a cytological assay recommended by the ASCCP for triage, has demonstrated a sensitivity of approximately 89.5% for HSIL+, though its specificity is often limited (around 47.2%), partly due to the subjective nature of cytological interpretation (33). In contrast, commercialized epigenetic assays have achieved higher specificities but show varying sensitivities; for instance, GynTect^®^ and QIAsure^®^ have reported sensitivity for HSIL+ at 59.0% and 65.0%, respectively, in independent validation cohorts (34). Furthermore, while emerging multi-target panels such as the S5 Classifier (targeting EPB41L3 and HPV types) (35) and the CISCER panel (PAX1/JAM3) have shown promising sensitivities of roughly 89.1% and 87.8% (36), our preliminary finding of nearly perfect sensitivity for HSIL in this pilot cohort suggests that single methylation site warrants further investigation as a highly sensitive alternative. Once again, the interpretation of these findings is primarily constrained by the single-center, retrospective design and the relatively small cohort size (total n=43), which may affect the generalizability of the results across diverse screening populations. Data from more rigorous, prospective cohorts are essentially needed to perform definitive head-to-head comparisons with current and emerging assays to validate the diagnostic edge of cg05057720 in a broader screening population.

Collectively, our findings hold significant potential for clinical application, as this novel biomarker demonstrates excellent detection performance for screening HSIL and cervical cancer patients, though larger multi-center studies are required to validate, further mechanistic studies are desired, and whether it works in more convenient methods like ctDNA screening warrants further exploration.

## Data Availability

The original contributions presented in the study are included in the article/**Supplementary Material**. Further inquiries can be directed to the corresponding author.
